# In Vitro Plant Evaluation Trial: Reliability Test of Salinity Assays in Citrus Plants

**DOI:** 10.3390/plants9101352

**Published:** 2020-10-13

**Authors:** Margarita Pérez-Jiménez, Olaya Pérez-Tornero

**Affiliations:** Equipo de Mejora Genética de Cítricos, Instituto Murciano de Investigación y Desarrollo Agrario y Alimentario (IMIDA), 30150 Murcia, Spain; margarita.perez3@carm.es

**Keywords:** *Citrus macrophylla*, mutagenesis, plant breeding, rootstocks, tissue culture

## Abstract

Salinity is one of the major abiotic stresses affecting crops worldwide, and breeders are urged to evaluate new genotypes to know their degree of tolerance to this selective agent. However, obtaining a number of plants high enough to make the evaluation can prove to be a long and laborious process which could be overcome by using tissue culture techniques. In the present study, the reliability of tissue culture evaluations is called into question through two parallel experiments, in vitro and ex vitro, using *Citrus macrophylla* and four mutants thereof, previously selected by their different behavior to salinity, as a plant material. Plants were subjected to salinity for 8 weeks in both in vitro (80 mM NaCl) and ex vitro (100 mM NaCl) experiments, and differences with plants grown in control conditions without salt were analyzed. After the experiments, length, leaf damage, shoot dry weight, chlorophylls and ions were measured in both conditions and experiments. As a result, it was demonstrated that tissue culture is a reliable tool to determine whether a genotype is tolerant to salinity or not, since plants of the same genotype responded in a similar way to salinity in both experiments. Henceforth, in vitro evaluations can be employed to test genotypes in a very early stage and using very little time and space. However, genotypes that showed the biggest or lowest changes when cultured in salinity were not always the same in both experiments. Thus, only *ex vitro* experiments can be performed if the goal is to compare genotypes and see which genotype is the most or least resistant to salinity.

## 1. Introduction

The evaluation of the stress response in plants is a laborious process that can also be long and costly when it comes to woody plants. Trees have lengthy biological cycles, which implies prolonged time lapses until plants are produced for study, as well as evaluations that can last for the entire growing season or even more than one season [1]. This is of particular importance in plant breeding programs where thousands of plants are handled yearly and the staff and land are restricted. However, the long juvenile periods and cost of maintaining all the seedlings until they are grown trees have encouraged researchers to develop early studies in young plants to discard those genotypes which do not fit in the breeding goals. Thus, researchers minimize the cost and time of field evaluations.

In recent decades, tissue culture has arisen as a valid method for stress evaluation, since it offers the possibility of managing large populations in a limited space while allowing a more rigorous control of the environmental conditions [2,3]. However, there are tissue culture detractors who claim that owing to the controlled environment, the heterotrophic conditions and the lack of roots, this technique is unreliable for stress studies. In the meantime, this technique has been used by many researchers to analyze stresses such as salinity in woody plants, some of them with an unpublished further *ex vitro* aspect [4,5,6,7], which raised doubts about the reliability of the selection. Other authors have published some *ex vitro* results of their in vitro selections that, although they proved their in vitro selections were tolerant to salinity, did not validate the reliability of the in vitro studies [8,9,10]. Hence, an extensive study using the most important physiological markers in salinity [11] with several genotypes is needed to see if a proper selection can be made by tissue culture.

In this study, a reliable validation of in vitro evaluations is proposed using citrus plants, with five genotypes and four mutants of *Citrus macrophylla*, previously selected by their different behavior to salinity and *Citrus macrophylla* plants being tested both in in vitro and ex vitro conditions. The study goal was to figure out if plants showed the same response to salinity in both in vitro and ex vitro conditions, their degree of tolerance when compared to the rest of the material and if results matched in both conditions.

## 2. Results

**Figure 1:**
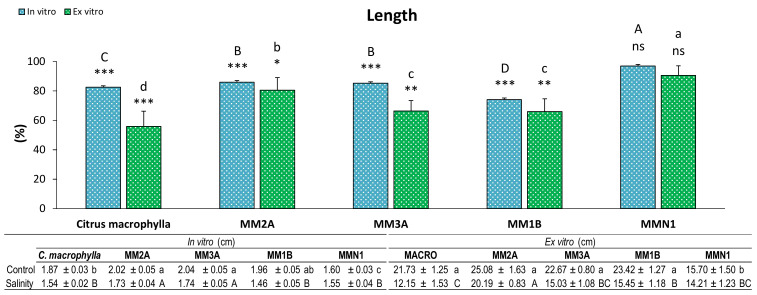
Percentage (%) of length of plants of *Citrus macrophylla* and the mutants MM2A, MM3A MM1B and MMN1 grown in salinity with respect to control conditions. Values are means ± SE. Capital letters indicate differences (*p* < 0.05) between genotypes in the in vitro experiment. Lower case letters indicate differences (*p* < 0.05) between genotypes in the ex vitro experiment. * Indicates the level of significance between saline and control conditions in each experiment (*, *p* < 0.05; **, *p* < 0.01; ***, *p* < 0.001). Data below the figure indicate means ± SE obtained in both control and salinity conditions. Capital letters indicate differences (*p* < 0.05) between genotypes in salinity conditions, lower case letters show differences in control conditions.

Length was reduced in both experiments because of salinity. Nearly all of the genotypes showed shorter lengths in salinity than in control conditions, with the exception of MMN1, which had similar growths in both salinity and control conditions in the in vitro and ex vitro experiments (Figure 1). However, while in the ex vitro experiment the genotype which had its length reduced the most was *Citrus macrophylla* (where length was almost halved), in the in vitro experiment MM1B was the genotype that had its length reduced the most (25.93%).

**Figure 2:**
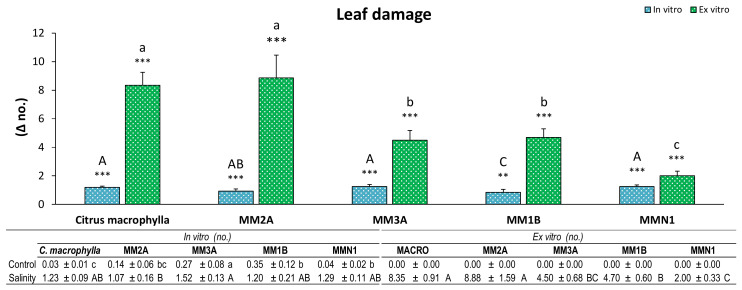
Increment of damaged leaves in plants of *Citrus macrophylla* and the mutants MM2A, MM3A MM1B and MMN1 grown in salinity with respect to control conditions. Values are means ± SE. Capital letters indicate differences (*p* < 0.05) between genotypes in the in vitro experiment. Lower case letters indicate differences (*p* < 0.05) between genotypes in the ex vitro experiment. * Indicates the level of significance between saline and control conditions in each experiment (*, *p* < 0.05; **, *p* < 0.01; ***, *p* < 0.001). Data below the figure indicate means ± SE obtained in both control and salinity conditions. Capital letters indicate differences (*p* < 0.05) between genotypes in salinity conditions, lower case letters show differences in control conditions.

Damage produced by salinity in the leaves was observed in all of the genotypes in both experiments (Figure 2). Nevertheless, while in the in vitro experiment almost all of the genotypes (except for MM1B, which showed a lower damage) had a similar increment of leaf damage, *Citrus macrophylla* and MM2A had double the number of damaged leaves than the rest of the genotypes in the *ex vitro* experiment.

**Figure 3:**
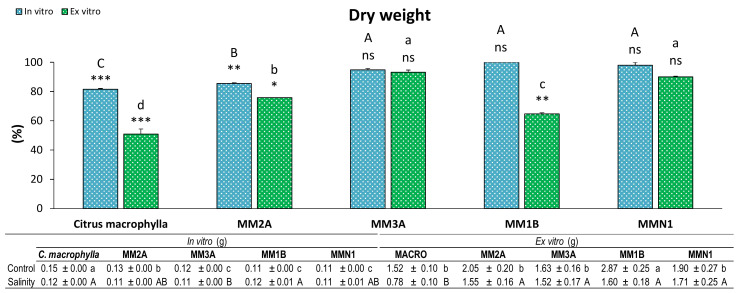
Percentage (%) of dry weight of the shoot in plants of *Citrus macrophylla* and the mutants MM2A, MM3A MM1B and MMN1 grown in salinity with respect to control conditions. Values are means ± SE. Capital letters indicate differences (*p* < 0.05) between genotypes in the in vitro experiment. Lower case letters indicate differences (*p* < 0.05) between genotypes in the ex vitro experiment. * Indicates the level of significance between saline and control conditions in each experiment (*, *p* < 0.05; **, *p* < 0.01; ***, *p* < 0.001). Data below the figure indicate means ± SE obtained in both control and salinity conditions. Capital letters indicate differences (*p* < 0.05) between genotypes in salinity conditions, lower case letters show differences in control conditions.

Dry weight of the shoot was measured after both experiments, and a significant loss of biomass by salinity was found in *Citrus macrophylla* and MM2A both in vitro and ex vitro (Figure 3). MM3A and MMN1 showed non-significant differences between control and salinity in the two experiments. On the other hand, although *Citrus macrophylla* was the genotype that decreased its dry weight the most and MMN1 and MM3A were the genotypes that decreased their biomass the least in both experiments, there is a discrepancy between results obtained by MM1B. In this genotype, dry mass decreased significantly in the experiment performed ex vitro, while no significant differences were found between control and salinity in the experiment performed ex vitro.

**Figure 4:**
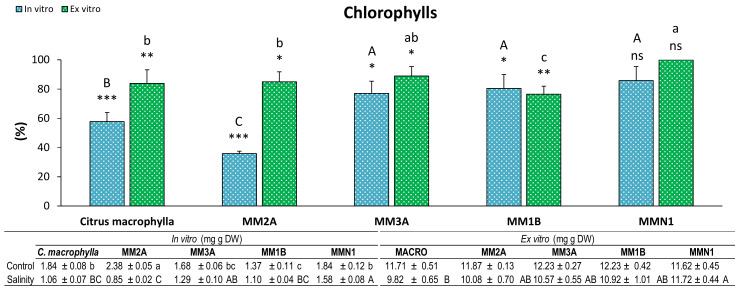
Percentage (%) of chlorophylls in leaves of plants of *Citrus macrophylla* and the mutants MM2A, MM3A MM1B and MMN1 grown in salinity with respect to control conditions. Values are means ± SE. Capital letters indicate differences (*p* < 0.05) between genotypes in the in vitro experiment. Lower case letters indicate differences (*p* < 0.05) between genotypes in the ex vitro experiment. * Indicates the level of significance between saline and control conditions in each experiment (*, *p* < 0.05; **, *p* < 0.01; ***, *p* < 0.001). Data below the figure indicate means ± SE obtained in both control and salinity conditions. Capital letters indicate differences (*p* < 0.05) between genotypes in salinity conditions, lower case letters show differences in control conditions.

The amount of chlorophylls was lower with salinity in both experiments in all of the genotypes, except for MMN1 (Figure 4). MM2A was the genotype which was affected the most by salinity in the in vitro experiment, whereas MM1B, MM2A and *Citrus macrophylla* displayed a higher decrease in the chlorophyll content of their leaves in the ex vitro experiment.

**Figure 5:**
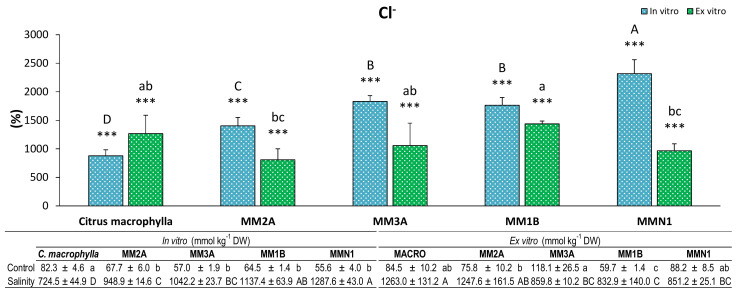
Percentage (%) of chloride (Cl^−^) in leaves of plants of *Citrus macrophylla* and the mutants MM2A, MM3A MM1B and MMN1 grown in salinity with respect to control conditions. Values are means ± SE. Capital letters indicate differences (*p* < 0.05) between genotypes in the in vitro experiment. Lower case letters indicate differences (*p* < 0.05) between genotypes in the ex vitro experiment. * Indicates the level of significance between saline and control conditions in each experiment (*, *p* < 0.05; **, *p* < 0.01; ***, *p* < 0.001). Data below the figure indicate means ± SE obtained in both control and salinity conditions. Capital letters indicate differences (*p* < 0.05) between genotypes in salinity conditions, lower case letters show differences in control conditions.

**Figure 6:**
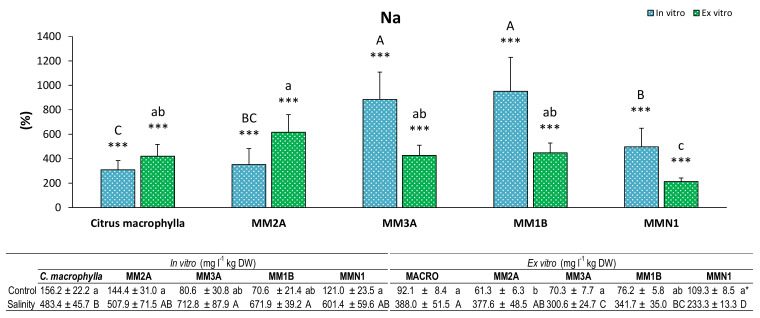
Percentage (%) of sodium (Na) in leaves of plants of *Citrus macrophylla* and the mutants MM2A, MM3A MM1B and MMN1 grown in salinity with respect to control conditions. Values are means ± SE. Capital letters indicate differences (*p* < 0.05) between genotypes in the in vitro experiment. Lower case letters indicate differences (*p* < 0.05) between genotypes in the ex vitro experiment. * Indicates the level of significance between saline and control conditions in each experiment (*, *p* < 0.05; **, *p* < 0.01; ***, *p* < 0.001). Data below the figure indicate means ± SE obtained in both control and salinity conditions. Capital letters indicate differences (*p* < 0.05) between genotypes in salinity conditions, lower case letters show differences in control conditions.

In terms of the ions which composed the salt, both of them (Na and Cl^−^) sharply increased in both experiments in all of the genotypes in salinity conditions with regard to control (Figure 5 and Figure 6). MMN1 was the genotype in which Cl^−^ content increased the most ex vitro, whereas in the ex vitro experiment MMN1 and MM2A exhibited the lowest increase (Figure 5).

On the other hand, MM1B and MM3A were the genotypes that increased their Na content the most in the in vitro experiment, and MM2A was the genotype that accumulated the highest amount of Na in salinity in the ex vitro experiment (Figure 6). The lowest contents were detected in *Citrus macrophylla* in the in vitro experiment and in MMN1 in the ex vitro assay.

## 3. Discussion

So far, many salinity screenings and studies have been performed without any validation. In this study, tissue culture has been proved to be a reliable tool to determine whether a genotype is sensitive to salinity or not, then in vitro studies are reliable and useful when we want to select genotypes that are tolerant to a certain selective agent. In this way, we can discard genotypes at a very early stage and using very little time and space. This fact is supported by data obtained in this experiment, where changes observed in a given marker in salinity compared to control conditions in the in vitro experiment meant the same kind of changes in the same marker in the ex vitro experiment. There is an exception to this statement, which is MM1B dry shoot weight. This uniqueness can be explained by the vigor that this genotype showed ex vitro, with a high multiplication rate in both control and salinity conditions (data not shown). As previously reported in pear [12], the high number of new shoots disguises the decrease in dry weight; consequently, special care should be taken when using this marker in in vitro experiments for study salinity tolerance.

Nevertheless, the in vitro experiment has failed in its attempt to compare the degree of sensibility/resistance of the different genotypes, which means that the most affected genotype in the in vitro experiment for a certain marker rarely matches the most affected genotype in the ex vitro experiment. Thus, tissue culture would be useful for choosing genotypes that are tolerant to a certain selective agent [8,10,13], but not to compare the degree of tolerance of the different genotypes among them. Then, if our aim is to see which genotype is the most or least resistant, tissue culture is not the tool. Therefore, the physiological mechanisms involved in salt tolerance would be better studied in ex vitro conditions. This is in contrast with studies in cassava [9], where, although differences between the in vitro and ex vitro experiments were observed amongst clones, the in vitro experiment correlated with the ex vitro assay.

Plants exposed to salinity undergo two different stages: a first phase in which osmotic pressure increases, making it harder for roots to extract water and producing dehydration symptoms in the plant; and a second phase, in which Na^+^ and Cl^−^ uptake greatly increases, and the accumulation of these ions at toxic concentrations leads to leaf damage and delayed growth [14]. Plants in the in vitro experiment were deprived from roots; therefore, cells of the basal part of the stem could be taking up mineral nutrients directly from culture media [15]. However, as leaf damage and shoot elongation indicated, the absorption would be produced in a different way than in real roots, given the divergent results of MM3A and MMN1 in leaf damage and Citrus macrophylla, or MM3A and MM1B in length, when compared to the rest of the genotypes in both experiments.

Roots are suggested to regulate toxic ion absorption through the modulation of hydraulic conductance and transpiration rate [9]. However, this lower absorption, whose aim is to accumulate less ions, would not be affecting the performance of MMN1, which already had a very good performance ex vitro. Then, the presence of roots in the ex vitro experiment only confirmed its potential ability to grow in saline environments giving more specific weight to the tolerance of the canopy than to the ability of the root to exclude ions. In contrast, MM1B had a worse performance in the ex vitro experiments when it was provided with roots, since it relatively accumulated more Cl^−^ and Na in salinity in the ex vitro experiment when compared to the rest of the genotypes, which led to a higher chlorophyll degradation [16].

Leaf damage is a parameter that showed remarkable differences in both experiments. Disparities between both conditions (control and salinity) obtained in vitro were minimal, while in the ex vitro experiment big differences between conditions were found. These differences were not that evident in the ion accumulation; therefore, leaf damage must be more related to the other effect of salt: osmotic stress [14]. Tissue culture plants grow in an environment rich in nutrients and relative humidity, which could attenuate the osmotic effect of salt. Anyway, differences are shown between both conditions in all genotypes, which demonstrates the validity of this parameter for the evaluation of genotypes in salinity conditions [11].

## 4. Materials and Methods

### 4.1. Experiment Design

#### 4.1.1. In Vitro Experiment

Plant material was collected from multiplication cultures of “Alemow” (*Citrus macrophylla*) citrus rootstock and its derived mutants (MM2A, MM3A, MM1B and MMN1) obtained in previous experiments [17] and selected by their different response to salinity, with regard to “Alemow”. Salinity stress was achieved by placing shoot tips (1–1.5 cm long) from the proliferation medium in each standard proliferation medium supplemented with 0 and 80 mM of NaCl. The level of salinity was chosen in previous studies of sensitivity to NaCl [18]. Each treatment consisted of six replicates (jars) with 12 shoots per jar. Explants were transferred into fresh medium every 4 weeks. After 8 weeks, samples were collected and shoot length, number of proliferating shoots longer than 5 mm per explant, number of leaves with damage per explant and dry weight were recorded. Additionally, leaves were collected for further analysis.

#### 4.1.2. Ex Vitro Experiment

Sixteen plants from an in vitro culture of each genotype, the same used in the in vitro experiment, were acclimatized and grown in 0.5 L black containers filled with peat in a climate chamber with fully-controlled environmental conditions: 60% RH, 16/8 h day/night at 25 °C. Plants were watered regularly with standard Hoagland solution supplemented with 0 and 100 mM of NaCl for 8 weeks (the level of salinity was chosen in previous studies of sensitivity to NaCl). Excess solution was drained from the pot to avoid salt accumulation in the substrate. After the salinity period, samples were collected and shoot length, number of damaged leaves and dry weight were measured. Some leaves were also collected for further analysis.

### 4.2. Chlorophyll Content

Chlorophyll content was estimated following the procedure described by [19], extracting 20 mg of ground material with *N*,*N*-dimethylformamide and measuring absorbance at 664.5 and 647 nm.

### 4.3. Ion Determination

Plant material was lyophilized and finely ground for analysis. After calcination at 550 °C, Na level was determined by inductively coupled plasma-optical emission spectrometry (ICP-OES) (Varian Vista-MPX, Varian Australia, Mulgrave, Vic., Australia). Cl^−^ was extracted from ground material (0.4 g) with 20 mL of deionized water. Samples were analyzed in an ion chromatograph (METROHM 861 Advanced Compact IC; METROHM 838 Advanced Sampler) using a METROHM Metrosep A Supp7 250/4.0 mm column.

### 4.4. Statistical Analysis and Data Presentation

Data were first tested for homogeneity of variance and normality of distribution. Significance was determined with an analysis of variance (ANOVA), and the significance (*p* < 0.05) of any differences between mean values was tested with Duncan’s New Multiple Range Test, using Statgraphics Centurion^®^ XVI (StatPoint Technologies Inc., The Plains, VA, USA) to do so.

Changes between control and salinity conditions in both experiments were expressed as a percentage of the value corresponding to plants grown in saline conditions with respect to plants grown in control conditions in both experiments, in vitro and ex vitro, or the increment of the value in saline conditions with respect to control when percentage calculation could not be performed (leaf damage). In addition, the level of significance between control and saline conditions was indicated for each genotype and experiment.

## 5. Conclusions

In summary, in vitro experiments have proved to be as reliable as *ex vitro* experiments for the search and study of citrus genotypes that are tolerant and sensitive to salinity. However, it is not possible to compare genotypes amongst them and, therefore, choose the most or least tolerant, since differences detected between genotypes in vitro do not correlate with differences between genotypes in the ex vitro experiments. Thus, the tissue culture methods likely need optimization for the real evaluation of salinity tolerance. This must be due to the role played by roots. However, this study demonstrates that salinity resistance is not only a matter of roots, since different results were obtained in their presence. Roots enhanced, worsen or did not change the performance of certain genotypes in the ex vitro experiment when compared with ex vitro. Hence, the canopy must be playing a major role on NaCl tolerance in citrus.
